# Input From Practice: Reshaping Dental Education for Integrated Patient Care

**DOI:** 10.3389/froh.2021.659030

**Published:** 2021-03-15

**Authors:** R. Lamont (Monty) MacNeil, Helena Hilario

**Affiliations:** University of Connecticut School of Dental Medicine, Farmington, CT, United States

**Keywords:** integration, dentistry, medicine, health systems, interprofessional education, dental education

## Abstract

Among the primary challenges in advancing the practice of integrated primary dental and medical health care is the appropriate educational and clinical preparation of a dental workforce that can function and flourish within integrated care environments. Most dental schools teach to traditional concepts and standards of dental care delivery which may be inconsistent with those of integrated care and could deter the entry and retention of graduates in contemporary, non-traditional practice models. To better understand how the dental school curriculum should be modified to accommodate integrative care models, a number of patient care organizations actively engaged in dental-medical integration were site visited to gain insight into the readiness of newer graduates, with emphasis on the US DMD/DDS graduate, to function in integrated practice. Leaders, practicing clinicians and staff were interviewed and common observations and themes were documented. This manuscript will focus on those educational components that integrated care organizations identify as absent or inadequate in current dentist education which must be addressed to meet the unique expectations and requirements of integrated patient care. These changes appear pivotal in the preparation of a dental clinician workforce that is respectful and receptive to new practice concepts, adaptative to new practice models, and competent in new care delivery systems.

## Introduction

Predoctoral (i.e., pre-DMD/DDS degree) dental education programs in the United States are in a perpetual mode of revision and adjustment as they attempt to respond to evolving change in society's needs and the practice of general dentistry [1–3]. Although these changes tend to emerge gradually, they are nevertheless real and compel dental schools to reconsider the knowledge, skills, abilities and values, collectively termed “competencies,” required by new dental graduates [4]. There are many examples of change in dental practice: shifts in care emphasis from a repair/restoration focus to prevention and early interceptive treatment; growth in multi-provider group and corporate-affiliated practice; increased focus on the social determinants of health; employment of evidence-based decision making in treatment planning; and greater attention on the relationship of oral and overall systemic health. The emergence of new models of care delivery is linked to many of these drivers of change and is perhaps best exemplified in the growth of integrated dental-medical care practice [5].

Some of the changes pursued by dental schools have been self-inspired but many have been prompted by the advocacy of thought leaders and national dental organizations to instill necessary updates in prevailing dental accreditation standards [6–8]. In response to professional and community input, the Commission on Dental Education (CODA) has introduced a number of new standards over the past several decades which, in turn, have prompted dental schools to modify curriculum, clinical practices and community interaction [9]. Several newer standards were informed by the embryogenesis of integrated dental-medical care and the opinion held by many in the profession that its continued development in both traditional and non-traditional models of practice could significantly improve health care outcomes [10, 11]. The following are examples of modified or new accreditation standards that refer to those competencies considered essential in integrated care delivery:

- Standard 2-15: *Graduates must be competent in the application of biomedical science knowledge in the delivery of patient* care^1^.
- Standard 2-19: *Graduates must be competent in applying the basic principles and philosophies of practice management, models of oral health care delivery and how to function successfully as the leader of the oral health care team*.
- Standard 2-20: *Graduates must be competent in communicating and collaborating with other members of the healthcare team to facilitate the provision of healthcare*^2^.

These and other accreditation standards and intent statements have led to the adoption of new educational approaches in US dental schools. One major initiative has been the development of interprofessional education (IPE) activities wherein dental students at timepoints within the typical four-year DMD/DDS curriculum are brought together in learning experiences with students from the other health professions including but not limited to medicine, nursing and pharmacy [12–14]. The objective is that these students learn together, better understand the relationship with and scope of other health professions, develop interprofessional communication skills and, optimally, co-participate in the coordinated care of patients [15]. Beginning in 2009, several health professions education organizations including the American Dental Education Association (ADEA) began to formally organize around these initiatives leading to the creation of the Interprofessional Education Collaborative, or IPEC, which currently includes 21 national health profession associations. In 2011, the first IPEC competencies were adopted [16], expanding in 2016 to four core competencies described by IPEC as essential for students in the health professions to succeed in interprofessional collaborative practice [17].

Beyond these particular changes, recommendations continue to be voiced on how dental schools can best respond to changes in the needs of society and the emergence of new healthcare systems and models of practice [4]. Schools have been moderately successful in analyzing their success in the implementation of curricular revisions but much less so on the impact of these responses on the preparedness of new graduates to function and succeed within new models of dental care especially those characterized by high levels of interprofessional interaction such as that observed in integrated dental-medical practice [18, 19].

Consequently, this project was undertaken to gain input from leaders of dental care entities with high levels of integrated care activity about the readiness of new DMD/DDS graduates for this unique form of practice.

## Methods

### Study Construct

During 2018-2019, the American Dental Education Association (ADEA), under the direction of its Chair of the Board of Directors, initiated the project with the goal to gain a better understanding about how US dental education institutions were currently preparing graduates for integrated care practice and possible areas where improvement in curriculum and/or clinical training was necessary [20]. As an initial step, the authors sought to identify dental practices and health care organizations that were actively engaged in a meaningful level of integrated care activity. The SAMHSA-HRSA Center for Integrated Health Solutions (CIHS) framework for levels of integrated health care [21] served as a reference to assess level of integrated care. Potential practices were identified by conducting literature searches of peer- reviewed publications and abstracts and by scanning conferences proceedings, meeting presentations, professional monographs and marketing materials. Entities appearing to have a moderate-to-high degree of integrated care activity and approaching CIHS's Levels 4-6 of collaboration/integration were targeted (i.e., Level 4- Close collaboration onsite with some systems integration; Level 5- Close collaboration approaching integrated practice; Level 6- Full collaboration in a transformed/merged integrated practice). In total, thirteen entities were identified and then contacted directly for further review. Two entities did not respond, one expressed a low level of interest in the project and three others were considered to have a relatively small amount of integrated activity. Based on initial response and interest in the project, seven entities remained and agreed to participate.

Each practice organization was site visited over a period of 1–2 days by one of the authors (RLMN) and included interactions with directors, care providers and support staff.

In the case of multi-site practices, a representative number of satellite locations were visited along with the primary site of care delivery. Structured questions (Table 1) were proposed to the chief administrative leaders. Interviews with clinical providers and auxiliary staff were less formal and more conversational in nature.

**Table 1:** Interview guide for integrated care practice leaders.

1. Describe (in common language) the key characteristics of your oral health care delivery approach/model and how it differs from a traditional (large) group dental practice?
2. Was your organization integrated from its inception? If so, what factors drove that integration? If not, when did your organization or group become interested in integration or greater connectivity with the larger health system and why? What were your key drivers? Who were your key “connected” health partners?
3. How are you connected to your other healthcare colleagues, and how do functionally communicate with them?
4. In terms of your initial goals, at what phase are you in your integration efforts?
5. At your current and unique phase of integration or connectivity, what do you see as the major, unique advantages of your delivery approach?
6. How have your integration efforts benefited your patients?
7. How have your integration efforts benefited the dental providers and staff?
8. How have your integration efforts benefited your other health care colleagues?
9. Are the competencies/skills needed by dentists in this system different than that needed in a traditional group practice? If so, what must dental providers bring to your practice approach in order to be successful within it?
10. How can dental academic institutions better prepare their graduates to be successful in organizations such as yours?
11. What advice can you provide to dental schools or academic health centers considering a more integrated approach in their clinical endeavors?

The focus of the interviews and interactions was to determine how the participants viewed the preparedness of recent dental school graduates, specifically dentists, for professional activity within an active, integrated care environment, and where gaps in the educational process were evident. “Recent graduate” was defined as a dentist graduating from a US DMD/DDS degree program within the past 5 years. The site visit also served to assess the level of integrated dental-medical care by the selected entity.

The seven care groups visited included two large managed care/HMO type organizations, a multi-site hospital/federally qualified health center (FQHC) care entity, a hospital-based system with a general practice residency program serving as its chief dental care arm, a benefits organization with an expanding care delivery network, and two large, multi-provider dental group practice. Sites spanned the Northeast, Midwest and Western region of the country. One site proved to have rather minimal integrated care activity, one declined to have its interview reports published, and two sites showed some integrated activity but substantially less than that of the three sites eventually selected as the key informants for the ADEA Association Report.

The three entities selected for reporting were Permanente Dental Associates (PDA) in Portland Oregon, Marshfield Clinic Health System in Marshfield, Wisconsin and HealthPartners in Minneapolis, Minnesota. The organizational construct, care philosophies and other characteristics of these three organizations are fully described in previous publications [22–24]. Following the site visits with these entities, summary notes taken by (RLMN) were shared with those interviewed to confirm accuracy. Using an inductive approach, responses to interview questions were grouped into common themes for further consideration. Conclusions and recommendations relative to new dentist preparedness and other aspects pertinent to dental education were then drafted, shared and finalized. A full description of these interviews and recommendations has been previously reported in a five-part Special Report in the Journal of Dental Education [22–26].

## Results/Discussion

### General Observations

In the initial scan of engaged sites, only a limited number were found to have meaningful and sustained integrative care activities with medical units or other health professions providers. A number of practices or organizations reporting integrated care were found to be practicing it at very low functional levels. Some were still in the planning stages while others were at the very early stages of implementation. This finding suggests that the dental profession is still very much at the embryonic stage of integrated practice. The apparently low number of active, functional sites poses certain limitations and challenges to dental education.

As the project got underway, several large multi-provider dental practices falling into the general description of dental service organizations, or DSOs, began reporting on increased engagement in integrated dental- medical care. At least one DSO practice reported that it had moved to better support its integration efforts through conversion of its dental electronic health record (EHR) to a fully integrated, nationally recognized EHR used by a large number of US hospitals and medical care networks [27]. Unfortunately, the current project was not able to include these organizations and their engagement could potentially have expanded the site visit pool and the diversity of perspectives gained.

### Perspectives and Recommendations From the Field

The following is a summary of the most common findings and suggestions gained through site visits at the three organizations described above and reported in greater detail in a special report of the Journal of Dental Education [22–26].

#### Interprofessional Education Must Be Improved Through Reinforcing Clinical Experiences

Organization leaders reported little difference in the preparedness of recent dental school graduates for integrated care practice compared to providers joining their organizations who graduated much earlier or who were engaged in prior traditional practice (“Recent graduate” was defined as a graduate of a dental school within the previous 5 years, between 2013 and 2018). This perspective was unanticipated as more recent graduates were expected to have had greater exposure to aspects of interprofessional education during their dental school training. Those interviewed were aware that dental schools had increased their emphasis on IPE in response to new accreditation standards and increased educational emphasis on IPE over the last decade. In general, they failed to see how that experience was translating into a different type of new provider or one more prepared for integrated care. Concern was expressed that IPE might be occurring in what one leader described as an “educational silo” [28], not strongly linked with active patient care and reinforcing clinical experiences, more formally termed interprofessional collaborative care (IPCC).

Based on the considerable investment that many schools have made in IPE, these comments should evoke reaction within the educational community. While the goals and objectives of IPE extend far beyond readying graduates for specific care environments, preparedness for delivery of integrated care represents a unique opportunity to measure IPE's impact in the form of a practical outcome. A strong recommendation from those interviewed in this project was that the IPE experience in dental schools not occur too distant or detached from the clinical practicum and that a good proportion of IPE should be embedded within clinical experiences where a measure of interprofessional collaborative care (IPCC) is practiced. This may be difficult for many schools with traditional intramural clinical operations, not part of an academic health center construct or not affiliated with other health professional schools or care units. Even for the approximate two-thirds of dental schools that are part of academic health centers, many have found it difficult to introduce and blend collaborative care activities with other health professions on campus. In some cases, it may be more feasible for dental schools to seek out extramural clinical sites where collaborative integrated care is active and could be modeled. One major limitation as previously noted is the relative paucity of dental settings where integrated care is active and institutionally supported.

#### Hospital- or Medically-Focused Residency Training Improves Preparedness

A correlate recommendation from those interviewed was that new providers with interest in integrated care practice should pursue advanced training of at least 1 year in a hospital-based general practice or pediatric dentistry residency program, representing the two chief elements of primary dental care. Graduates of general practice residency (GPR) programs that are closely aligned with hospital or medical operations and which provide coordinated care for inpatient populations were viewed as distinguishable from other new dental providers. The respondents felt that this was likely the result of the professional interactions between dentistry, medicine and the other health professions required within these types of programs. Not surprisingly, all three organizations had moved to placing substantial emphasis on a GPR experience as part of new provider recruitment. One organization felt that graduates of a particular US dental school were much better prepared for their brand of integrated practice; this particular school places senior students in extended community health center rotations and in several different locations nationally during the majority of the fourth year of their DMD/DDS education. This organization felt that these experiences exposed students to the dynamics of oral health inequities, health care disparities, economic/social/cultural determinants of health, and the treatment of acute dental disease, all which were an important part of their operational mantra. A general recommendation was that dental schools should sustain, if not expand, the amount of clinical time spent at community-based sites and, if possible, at FQHCs where medical and dental units support an integrative care philosophy and approach.

#### Maintain a Strong Curricular Experience in the Medical Sciences but Ensure an Applied, Practical Focus

The interviews identified a concern that dental schools were downscaling their basic and applied medical science curriculum in favor of competing curricular interests. The following examples were articulated: schools providing students with earlier clinical experience by transferring curricular time traditionally devoted to medical science instruction to the clinical practicum; a greater proportion of the available curriculum now devoted to exposure to and instruction in new chairside and laboratory technologies with focus on dental procedures; a fast-tracking of students through the core medical curriculum to meet the standards set by national boards but insufficient to provide the depth of knowledge needed for application in patient care; within the medical training of dental students insufficient emphasis placed on the discreet number of systemic conditions and diseases closely linked with oral health and provision of dental care. Some interviewed suggested that the dentist should be trained to the same level as primary care physicians relative to the limited number of medical conditions dominant within primary care and that consume the majority of integrated care communication and co-therapy planning such as diabetes, hypertension, and asthma. There was a perception that new dental providers have superficial knowledge across a wide range of medical conditions yet inappropriate depth and application ability in these common conditions. A prevailing opinion was that the foundational and applied medical curriculum within dental education must remain strong with emphasis on the possible unique role of dentists in the co-management of common medical conditions and the effective closure of gaps in disease prevention and health promotion strategies. The design of this type of curriculum requires effective communication between leaders of dental and other health professional schools and the coordinated participation of the entire dental faculty spanning generalist to specialist.

#### Invest in Integrated Electronic Health Records Allowing Communication Between Dental Medicine and the Other Health Professions

The issue of improving upon methods of professional communication was the strongest recommendation from our participants. It was strongly recommended that dental schools move toward participation in electronic health records (EHRs) shared with the medical community including hospitals and physician networks [29, 30]. The functional divide created by dental medicine and primary care medicine working from separate, unconnected EHRs was felt to be dramatically detrimental overall, placing dental practice in isolation from the surrounding and changing healthcare world. Optimism was expressed that several US dental schools recently moved away from dentistry-only EHRs to more broadly used health care platforms. While an expensive and challenging endeavor, this change was seen as essential if integrated care is to grow and new dental providers are prepared to be actively engaged. Of note, the three organizations surveyed here each employ a unifying EHR with integrated medical and dental components.

#### Provide Practical Experience in the Use of Health Analytic Tools

Contributors stressed the importance of the modern dental care provider being versed in the use of health analytic tools. These tools are often supported by contemporary, integrated EHRs, yet another reason for student exposure to such records. It was anticipated that providers will be more motivated to pursue innovative care strategies if they can witness the results of these efforts in improved quality care metrics. One example was the demonstrated ability by one organization to close gaps in certain medical preventive care protocols by the engagement of the dental team [22]. Those interviewed expressed that dental providers must feel comfortable and confident in accessing these analytic tools and in interpreting the data.

#### Educate Students About New Dental Practice and Compensation Models

Questions were raised about the efficiency of dental schools in providing practice management concepts that vary from the traditional, prevailing fee-for-service, independent contractor model of dental practice. As integrated medical-dental care is further pursued, and as the dental profession shifts from a repair/restore emphasis to a more prevention/early disease interception mode, it is probable that other business models of practice will emerge. For example, it is predicted that group practices will likely grow in size and number [31] and more third-party plans will incorporate value-based reimbursement approaches. Within these changes, our interviewees predicted that outcomes-focused, incentive-based formulas tightly linked to the ability of the dental provider to practice in diverse teams that include non-dental professionals will capture a larger part of the compensation landscape. Dental students must be exposed to these concepts so that as new graduates they can understand their relative strengths and weaknesses and make informed decisions on the type of practice that best matches their preferences and shifts in future dental care.

#### Stress the Power and Art of Effective Interprofessional Communication

A common thread across these interviews was the critical importance the individual provider to effectively communicate with all members of the care team in order to flourish in a more integrative model of care. There was a position that most schools could still improve upon training in both intraprofessional (i.e., within the dental team proper) and interprofessional communication. In a number of the practices visited, auxiliary personnel including dental hygienists, licensed practical nurses (LPNs) and medical assistants (MAs) performed a large portion of the integrated care effort including identification of medical/dental risk, gaps in care, and in the clinical provision of services such as blood pressure monitoring, blood draws and vaccine administration. The importance of effective communication between the supervising dentist and auxiliary staff is essential, as is the necessary interprofessional communication with correlate members of the primary care medical team. It was advised that increased efforts be made to prepare dental students to consistently and accurately use contemporary medical terminology to ensure the effective and safe transfer of information across the integrated team.

#### Engage Students in Highly Functioning, Intraprofessional and Interprofessional Teams, and Incentivize Effective Teamwork

An overwhelming recommendation from the interviews was that dental curricula further advance the concept of team-based care as essential to traditional and contemporary forms of practice. Students must be exposed to dynamic team environments where they practice as authentic members of a team in the care of patients. While simulated environments may be useful in this task, exposure to working examples of active teamwork was deemed critical. It was recognized, however, that identification of these examples may be difficult but must be pursued. Again, dental schools may need to move outside their intramural systems of care and explore external, community- based models to achieve this goal.

## Conclusion

**Figure 1:**
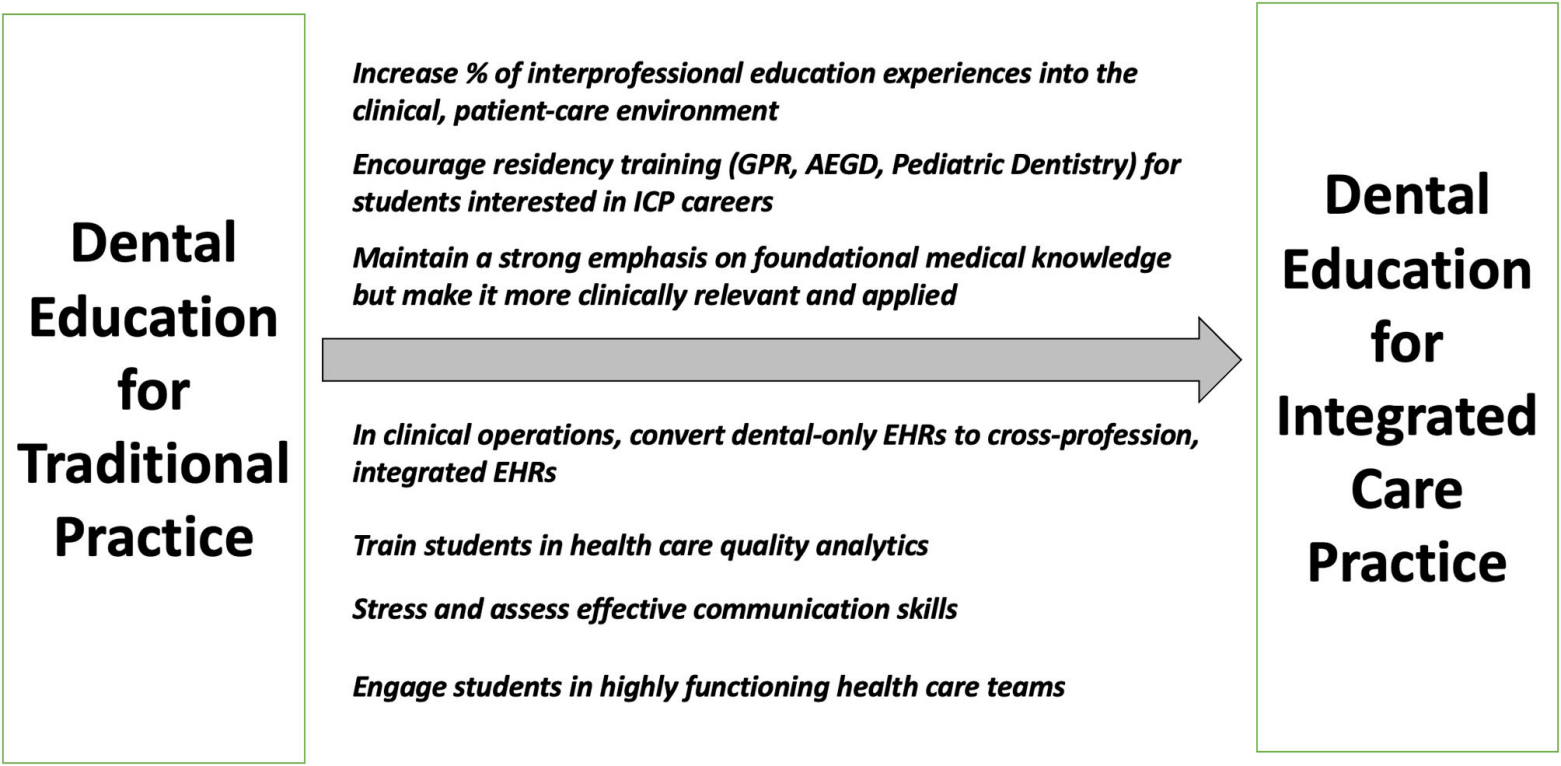
Integrated care practice leader recommendations for improvement in the education and preparedness of dentists for future practice.

The recommendations emanating from this interview survey with leaders of integrated care practices (summarized in Figure 1) suggest that despite efforts to adequately prepare graduates for interprofessional collaborative care, improvements are necessary. Institutional responses to recent accreditation standards and national initiatives such as those emphasizing interprofessional education have undoubtedly had an impact but perhaps not to the extent envisioned. The dental education community should consider the presented recommendations and continue to advocate for advancement of current programs and continual refinement in the guidelines and principles upon which future program development is propelled. This report suggests that interprofessional education must move to the forefront and that greater efforts be undertaken to identify examples of integrated practice in the community where interprofessional, collaborative care is actively and effectively modeled.

These recommendations may present additional challenge for dental education as it attempts to respond to and address the many diverse drivers and indicators for change in the dental curriculum. Several of these recommendations will not align easily or fluidly with other shifts currently being witnessed or suggested (see Fontana). For example, devoting more curricular time to applied medical science and interprofessional collaborative care could be viewed as incongruent with expansion and earlier introduction of clinical care hours devoted to traditional care and greater immersion in new dental technologies. Experimentation with collaborative team models where appropriate compensation strategies have not yet been developed may be deemed inconsistent with the urgency to create more sustainable intramural clinical care systems and improved net clinical revenues. Investments in expensive universal EHRs will undoubtedly prove difficult as dental institutions attempt to curtail or reduce the rising cost of dental education. Despite these challenges, the promise and possibilities found within integrated dental-medical care demands that it be given high priority in dental education [3, 32]. New strategies will most likely require non-traditional approaches, innovation and significant adjustment in the current educational model, and in particular, the clinical practicum [33]. Therein lies the challenge for today's dental schools and leaders in dental education.

## Data Availability Statement

The datasets generated in this article are not readily available because the dataset consists of narratives and notes collected from key informant site visits. Permission from the contributors was not attained for further distribution. Requests to access the datasets should be directed to macneil@uchc.edu.

## Ethics Statement

Ethical review and approval was not required for the study on human participants in accordance with the local legislation and institutional requirements. Written informed consent for participation was not required for this study in accordance with the national legislation and the institutional requirements.

## Author Contributions

All authors listed have made a substantial, direct and intellectual contribution to the work, and approved it for publication.

## Conflict of Interest

The authors declare that the research was conducted in the absence of any commercial or financial relationships that could be construed as a potential conflict of interest.

## Footnotes

^1^Each CODA standard is accompanied by an Intent Statement(s). The Intent Statement within standard 2-15 is that the graduate can “…*integrate new medical knowledge and therapies relative to oral health care*”.

^2^An Intent Statement within standard 2-20 is that students should “... *have educational experiences, particularly clinical experiences, that involve working with other healthcare professional students and practitioners. Students should have educational experiences in which they coordinate patient care with the healthcare system relative to dentistry*”.
